# Characterization of microglia/macrophage phenotypes in the spinal cord following intervertebral disc herniation

**DOI:** 10.3389/fvets.2022.942967

**Published:** 2022-10-03

**Authors:** Bianca Kühl, Martin Beyerbach, Wolfgang Baumgärtner, Ingo Gerhauser

**Affiliations:** ^1^Department of Pathology, University of Veterinary Medicine Hannover, Foundation, Hannover, Germany; ^2^Institute for Biometry, Epidemiology and Information Processing, University of Veterinary Medicine Hannover, Foundation, Hannover, Germany

**Keywords:** dog, immunohistochemistry, macrophages, microglia, spinal cord, spinal cord injury (SCI), spleen, trauma

## Abstract

Dogs frequently suffer from traumatic spinal cord injury (SCI). Most cases of SCI have a favorable prognosis but 40–50% of dogs with paraplegia and absence of nociception do not regain ambulatory abilities, eventually leading to euthanasia. Microglia and infiltrating macrophages play a crucial role in inflammatory process after SCI. However, little is known about microglia/macrophage phenotypes representing a potential target for future therapeutic strategies. In the present study, the microglia/macrophage phenotype was characterized by immunohistochemistry in the morphologically unaltered canine spinal cord (10 control dogs) and during acute and subacute SCI (1–4 and 5–10 days post injury, 9 and 8 dogs, respectively) using antibodies directed against IBA1, MAC387, MHC-II, lysozyme, EGR2, myeloperoxidase, CD18, CD204 and lectin from *Griffonia simplicifolia* (BS-1). The expression of these markers was also analyzed in the spleen as reference for the phenotype of histiocytic cells. Histological lesions were absent in controls. In acute SCI, 4 dogs showed mild to moderate hemorrhages, 2 dogs bilateral gray matter necrosis and 6 dogs mild multifocal axonal swellings and myelin sheath dilation. One dog with acute SCI did not show histological alterations except for few dilated myelin sheaths. In subacute SCI, variable numbers of gitter cells, axonal changes and dilated myelin sheaths were present in all dogs and large areas of tissue necrosis in 2 dogs. Neuronal chromatolysis was found in 3 dogs with acute and subacute SCI, respectively. In control dogs, microglia/macrophage constitutively expressed IBA1 and rarely other markers. In acute SCI, a similar marker expression was found except for an increase in MAC387-positive cells in the spinal cord white matter due to an infiltration of few blood-borne macrophages. In subacute SCI, increased numbers of microglia/macrophages expressed CD18, CD204 and MHC-II in the gray matter SCI indicating enhanced antigen recognition, processing and presentation as well as cell migration and phagocytosis during this stage. Interestingly, only CD204-positive cells were upregulated in the white matter, which might be related to gray-white matter heterogeneity of microglia as previously described in humans. The present findings contribute to the understanding of the immunological processes during SCI in a large animal model for human SCI.

## Introduction

Dogs frequently suffer from traumatic spinal cord injury (SCI) (1). A recent publication estimates 20.000 to 30.000 new cases presented to and managed by veterinary surgeons in the United States alone each year (2). SCI in dogs is most commonly related to intervertebral disc herniation (IVDH) subsequently to intervertebral disc degeneration (3, 4) causing a mixed contusive and compressive injury to the ventral aspects of the spinal cord (5–7). Other less common causes of SCI include traumatic fractures/luxation of vertebrae, fibrocartilagenous embolic myelopathy (FCEM)/ischemic myelopathy, acute non-compressive nucleus pulposus extrusion (ANNPE), compressive hydrated nucleus pulposus extrusion (HNPE) and intradural/intramedullary disc extrusion (IIVDE) (3, 8). Clinical signs of traumatic SCI are variable and range from back pain to complete loss of motor and sensory function impairing the quality of life of affected individuals (9). Due to advanced diagnostics, therapeutic options and high standards of clinical management, the outcome of mild to moderate cases of SCI in dogs is usually favorable. Unfortunately, 40–50% of dogs with severe SCI (paraplegia, absence of nociception) do not regain ambulatory abilities, eventually leading to euthanasia (10). Consequently, as valued companion animals, there is an increasing interest in improving therapeutic strategies (4, 10–12). Extensive research has been performed to understand the underlying pathological mechanisms using well-stablished rodent models. The initial traumatic insult in SCI is accompanied by shearing or disruption of axons, disturbances of axonal transport and impairment of blood flow. This primary damage initiates a cascade of secondary changes (secondary injury) including impairment of microcirculation, altered intracellular ion concentration, excitotoxicity, production of free radicals, and inflammation leading to widespread tissue destruction, which is even more deleterious than the initial insult (4, 13, 14). Fiber tract pathology is the central hallmark of various forms of central nervous system (CNS) injury (15) and a key feature of canine SCI, extending cranially and caudally beyond the epicenter (16) most likely leading to motor and sensory dysfunction (10). Despite the intensive bulk of information gained from rodent models, translation into successful clinical trials is often insufficient (5, 17, 18). Therefore, increased effort has been made to elucidate secondary inflammatory processes during naturally occurring SCI in dogs (19, 20).

Microglia play a crucial role in inflammatory processes after SCI in rodents, dogs and humans (19, 21–23). Microglia constitute a subset of CNS myeloid cells that make up for 5–12% of the total glial cell population (24) and together with non-parenchymal meningeal, perivascular and choroid plexus macrophages represent the innate immune cells of the CNS critically functioning in the maintenance of CNS integrity (25–27). In response to pathological stimulation, they shift in phenotype (26, 28) and are capable of antigen processing and presentation (29, 30), phagocytosis, modulation of T-cell response as well as production and release of cytokines, chemokines, nitrogen species and reactive oxygen species (31–33). Upon activation, they switch from a finely ramified (previously called “resting”) to a swollen ramified phenotype with stout processes (“activated”) and subsequently to a rod-shaped or round to oval morphology without processes (“reactive”) (28). Due to their ability of phagocytosis and the release of potentially cytotoxic substances, microglia/macrophages are considered to contribute to axonal damage and cell death by participating in the secondary auto-destructive mechanisms after SCI (27, 33). Increased functional recovery and neuroprotection can be achieved by depletion or functional inhibition of CNS macrophages after SCI in rats (34–36). On the other hand, they are also involved in tissue remodeling, repair and homeostasis by removal of cell debris and release of anti-inflammatory and wound-healing factors (27, 32, 37–40). An improvement of axonal regeneration was seen in rats with SCI following implantation of macrophages, which were pre-exposed *ex vivo* to peripheral nerve segments (41). This plurality of functions is reflected by a high phenotypic variability and microglia are able to simultaneously express pro- and anti-inflammatory markers *in vivo* (26, 32, 42, 43). Thus, the role of microglia/macrophages in SCI is regarded as a double-edged sword and still discussed controversially (31, 37, 44). The microenvironment in early SCI is characterized by pro-inflammatory conditions (19, 45–47) overrunning a weaker and transient anti-inflammatory microglia/macrophage response (48). Pro-inflammatory macrophages show increased phagocytosis as well as production and release of tissue degrading matrix metalloproteinases (MMPs), neurotoxic mediators and reactive oxygen species (ROS) causing collateral damage to healthy tissue (27, 49–51). Consequently, the polarization of microglia/macrophage toward a tissue sparing and regeneration promoting anti-inflammatory phenotype in CNS injury is regarded as a promising target for future therapies (37, 48, 52, 53).

Dogs gained increased attention as valuable bridging animal model mimicking certain conditions of the disease in humans more closely than conventional rodent models (7, 10, 16, 44, 54). Similar aspects of human and canine SCI comprise a relatively high incidence in the population, its spontaneous and natural occurrence, the nature of the injurious force, potential co-morbidities, individuality of patients, spinal cord size and certain aspects of pathology (7, 54, 55). Spontaneously occurring SCI is mostly elicited by structures located ventrally to the spinal cord such as herniated intervertebral disc contrasting rodent models in which the injurious force is placed to its dorsal aspect (7). Moreover, segmental demyelination in human and canine SCI seems to occur delayed (10, 16) and may progress to spinal cord cavitation, gliosis and spinal cord atrophy (21, 56–59), whereas in mice cavitation is not present and considerable scar formation can be found in chronic stages (22). Additionally, participation of lymphocytes in the immunologic processes seems to be subordinate in canine and human SCI (19, 21, 23).

Several studies investigated microglia/macrophages in healthy and injured canine spinal cord in *ex vivo* studies using flow cytometry. However, little is known about immunophenotype and morphological characteristics and their *in vivo* distribution within spinal cord lesions (44, 60). The aim of the present study was to investigate the microglia/macrophage phenotype in the canine spinal cord on the protein level focusing on the temporal aspect of SCI lesion development. Moreover, we studied the distribution of the used markers in the spleen of healthy dogs as reference for the phenotype of histiocytic cells.

## Materials and methods

### Tissue sampling

The study included paraffin-embedded spinal cord samples from 17 dogs with SCI and spinal cord samples from 10 control dogs without CNS lesions. Diagnosis of intervertebral disc herniation was based on clinical examination and imaging diagnostics including X-rays and magnetic resonance imaging (MRI) and confirmed by necropsy (Table 1). Samples were archived in the Department of Pathology from the University of Veterinary Medicine Hannover. Spinal cord samples of dogs with SCI and control dogs have been investigated in previous studies (16, 19, 61) and were studied for spinal cord microglia/macrophage occurrence and immunophenotype. Additionally, the spleens of five healthy beagle dogs were checked for macrophage occurrence, distribution and immunophenotype and served as a reference for the findings within the spinal cord. These beagle dogs have also been investigated in previous studies, which were approved by “Niedersächsisches Landesamt für Verbraucherschutz und Lebenmittelsicherheit”, Oldenburg, Germany (permission numbers: 33.9-42502-05-12A241 and 33.9-42502-05-13A346).

**Table 1:** Dogs used in the study.

**No**	**Case No**	**Group**	**Location of SCI**	**Time (days)**	**Age (years)**	**Sex**	**Breed**	Diagnostics*	**Treatment**
1	V 480/07	1	-	-	0.5	f	Beagle	-	-
2	V 481/07	1	-	-	0.5	f	Beagle	-	-
3	V 482/07	1	-	-	0.5	f	Beagle	-	-
4	S 1475/07	1	-	-	0.9	m	Labrador Retriever	-	-
5	S 1498/07	1	-	-	8	mn	Small munsterlander	-	-
6	S 1665/07	1	-	-	13	f	Dachshund	-	-
7	S 1682/07	1	-	-	10	f	Dachshund	-	-
8	S 913/09	1	-	-	14	m	Mongrel	-	-
9	S 1025/09	1	-	-	10	m	Pit bull terrier	-	-
10	S 1084/09	1	-	-	11.5	m	Golden Retriever	-	-
11	S 1449/07	2	Th10/Th11	4	9	m	Dachshund	Unknown	Unknown
12	S 1450/07	2	L2/L3	3.5	4	m	Dachshund mix	Unknown	NSAID/CS
13	S 563/08	2	Th12/Th13	3	7	f	Dachshund	X-ray; MRI	Surgery; NSAID/CS
14	S 1345/08	2	Th12/Th13	4	5	m	Dachshund	X-ray; MRI	Surgery; NSAID/CS
15	S 1378/08	2	C6/C7	1.5	12	m	Fox terrier	X-ray	None
16	S 99/09	2	Th13/L1	3	3.5	m	Pekinese	X-ray	Unknown
17	S 258/09	2	L2/L3	1.5	9	m	Mongrel	Unknown	Unknown
18	E 1999/09	2	L3/L4	1.5	7	m	Bernese mountain dog	X-ray; MRI	None
19	E 4043/09	2	L5/L6	1	5	m	Hanoverian scenthound	X-ray; MRI	NSAID/CS
20	S 1322/07	3	Th11/Th12	7	8	fn	Hanoverian scenthound	X-ray; MRI	Surgery; CS
21	S 1464/07	3	Th12/Th13	8	8	m	Dachshund	X-ray; MRI	CS
22	S 1638/07	3	Th11/Th12	10	7	m	Dachshund	X-ray; MRI	Surgery
23	S 1640/07	3	Th13/L1	7	4	m	Dachshund	X-ray; MRI	None
24	S 1549/08	3	C5/C6	>5	10	f	Dachshund	X-ray; MRI	Surgery; NSAID/CS
25	S 22/09	3	Th12/Th13	10	7	m	Mongrel	X-ray	NSAID/CS
26	S 75/09	3	Th12/Th13	8	10	f	Mongrel	X-ray	Unknown
27	E 4369/09	3	C2/C3	5	5	m	Dachshund	X-ray; MRI	Surgery
28	V 530/12	4	-	-	1	f	Beagle	-	-
29	V 532/12	4	-	-	1	f	Beagle	-	-
30	V 533/12	4	-	-	1	f	Beagle	-	-
31	V 836/13	4	-	-	2	m	Beagle	-	-
32	V 837/13	4	-	-	2	m	Beagle	-	-

No: number; Group 1: control group; Group 2: acute SCI (1–4 days); Group 3: subacute SCI (5–10 days); Group 4: healthy dogs used for investigation of spleen; C: cervical vertebra; CS: corticosteroids; f: female; fn: female neutered; L: lumbar vertebra; m: male; mn: male neutered; MRI: magnetic resonance imaging; NSAID: non-steroidal anti-inflammatory drugs; SCI: spinal cord injury; Th: thoracic vertebra.

Dogs for spinal cord investigation were divided into three groups. Group 1 included 10 control dogs without evidence of neurological diseases. Group 2 consisted of 9 dogs with a disease duration of 1–4 days (mean: 2.55 days; acute SCI). Group 3 contained 8 dogs with a disease duration of 5–10 days (mean: 7.58 days; subacute SCI). One dog with acute SCI showed tetraplegia. Clinical findings also included paraplegia in 7 out of 9 dogs with acute SCI and 4 out of 8 dogs with subacute SCI. Two dogs with subacute SCI showed loss of deep pain perception in addition to paraplegia. Two other dogs with subacute SCI exhibited neck/back pain. For one dog with acute SCI (No. 17) and two dogs with subacute SCI (No. 22 and 27) anamnestic data were not available. Eight of these dogs (No. 12–14, 19–21, 24, 25) received therapy with non-steroidal anti-inflammatory drugs (NSAID) or corticosteroids. Surgery was performed in 6 dogs (No. 13, 14, 20, 22, 24, 27). Seven dogs obtained no medical treatment. Data concerning previous treatment was not available from two dogs (No. 16 and 26). The dogs either died during anesthesia/surgery or were euthanized due to request of theirs owners. Necropsy was performed and a full organ spectrum including the entire spinal cord was collected. For further investigation, a 0.5 cm spinal cord sample was taken from the epicenter (macroscopically confirmed site of disc herniation) and fixed in 10% non-buffered formalin for four days. Finally, the five healthy beagle dogs used for histologic investigation of the spleen have been designated as group 4 (Table 1).

### Immunohistochemistry and lectin histochemistry

Immunohistochemistry of formalin-fixed and paraffin-embedded tissue was performed as described (62). Briefly, deparaffinized and rehydrated 4-μm sections were incubated in 85% ethanol with 0.5% H_2_O_2_ for 30 min at room temperature (RT) to block endogenous peroxidase. After pre-treatment (Table 2) background staining was blocked with inactivated goat serum diluted 1:5 in phosphate-buffered saline (PBS) except for slides labeled with BS-1. Primary antibodies (Table 2) were incubated overnight at 4°C. As negative control, monoclonal and polyclonal antibodies were substituted by ascites from BALB/c mice or rabbit serum (Sigma-Aldrich, Taufkirchen, Germany; R4505) respectively. Biotinylated goat-anti-mouse or goat-anti-rabbit IgG served as secondary antibodies (45 min, RT) or sections were labeled with biotinylated lectin (BS-1). Immunostaining was visualized using the avidin-biotin-peroxidase complex (VECTASTAIN Elite ABC Kit; Vector Laboratories, PK 6100, Burlingame, CA) method with 3,3'-diaminobenzidine-tetrahydrochloride-H_2_O_2_ (DAB; Sigma Aldrich) as chromogen. Finally, sections were slightly counterstained with Mayer's hemalaun.

**Table 2:** Antibodies used for immunohistochemistry.

**Antibody**	**Cell type detected**	**Type and Clone**	Dilution*	**Pretreatment**
CD18	bone-marrow derived leucocytes, dendritic cells	mAb (CA16.3C10; Prof. Dr. F. Moore)	1:10	proteinase K
CD204 (Macrophage scavenger receptor 1, MSR1)	tissue resident macrophages, vascular smooth muscle cells, endothelial cells	mAb anti-human CD204 (SRA-E5; Abnova)	1:500	MW
early growth response 2 (EGR2, KROX20)	neuroectodermal cells, macrophages	pAb anti-human EGR2 (aa397-446-HRP; LifeSpan BioScience, Inc.)	1:2000	-
ionized calcium-binding adapter molecule 1 (IBA1)	microglia/macrophages, vascular tissue, dendritic cells	pAb (019-19741; Waku)	1:500	MW
lectin from *Griffonia simplicifolia* (*Bandeiraea simplicifolia*, BS-1)	microglia/macrophages	Biotin-conjugated lectin (L3759-1MG; Sigma Aldrich)	5 μl antibody in 1 ml PBS	-
lysozyme	neutrophils, macrophages, body fluids (excl. urine and CSF), monocytes, glandular cells	pAb anti-human lysozyme (A0099; Dako)	1:4000	MW
major histocompatibility complex-II	antigen presenting cells	mAb anti-human MHC-II HLA-DR (TAL.1B5; Dako)	1:100	MW
myeloid/histiocytic antigen (L1)	neutrophils, monocytes, macrophages, epithelial cells	mAb anti-human myeloid/histiocytes antigen (MAC387; Dako)	1:500	MW
myeloperoxidase	monocytes, macrophages, neutrophils	pAb anti-human myeloperoxidase (ab9535; Abcam)	1:400	MW

^*^Dilution in phosphate-buffered saline with 1% bovine serum albumin. CD: cluster of differentiation; CSF: cerebrospinal fluid; mAb: mouse monoclonal antibody; MW: in citrate buffer (pH 6.0-6.5) and heated in microwave for 24 min; pAb: rabbit polyclonal antibody; PBS: phosphate-buffered saline.

For quantitative evaluation of the microglia/macrophage immunostaining in spinal cord transversal sections, the white matter was divided into six areas (dorsal, lateral and ventral funiculus; both sides) and the gray matter into two areas (left and right side). Microglia/macrophages were counted in four high power fields (HPF, 40x magnification) per area using a counting grid (counting area: 0.063 mm^2^). To restrict cell counting to microglia/macrophages, only immunopositive cells with a characteristic morphology of microglia/macrophages (ramified microglia: small cells with a small nucleus and abundant delicate ramified processes; rod-shaped microglia: small rod-shaped cells; round microglia/macrophages: small to medium-sized round to oval cells without ramified processes; gitter cells: medium-sized “foamy” round to oval cells with numerous lipid vacuoles) were included into the analysis. Immunostaining of macrophage markers in the spleen was evaluated using a semiquantitative score (0 = 0%; 1 = 1–25%; 2 = 26–50%; 3 = 51–75%; 4 = 76–100%). The white pulp [follicles, marginal zone, periarteriolar lymphoid sheaths (PALS)] and the red pulp (periarteriolar macrophage sheaths (PAMS), sinusoids/red pulp vascular spaces) of the spleen were scored separately. All splenic follicles in one section, 10 randomly chosen PALS and PAMS per dog and 10 randomly chosen high power fields of the sinusoids/red pulp vascular spaces were included into the evaluation. Pictures of stained sections were taken by an Olympus BX51 digital camera microscope [Olympus Optical Corporation [Europe], Hamburg, Germany] using the CellSens Standard 1.18 software (Olympus Corporation). Gnu Image Manipulation Program (GIMP) 2.10.22 software was used to enhance contrast and brightness.

### Statistical analysis

Statistical analysis was performed using GraphPad Prism version 9.0.0. Ink for Windows (GraphPad Software, Inc, La Jolla, CA). D'Agostino-Pearson test revealed lack of normal distribution of some data. Thus, non-parametric Kruskal-Wallis tests followed by Dunn‘s multiple comparisons tests or Friedman-test with Dunn‘s multiple comparisons tests were used to analyze spinal cord and spleen data, respectively. *P*-values below 0.05 were considered significant.

## Results

### Histology

No microscopic lesions were detected in spinal cord tissue from healthy control dogs. Four dogs with acute SCI showed mild to moderate hemorrhages (no. 12–15) predominantly restricted to the gray matter. In moderate hemorrhages (no. 13 and 14) also few neutrophils were detected. One dog (no. 18) showed a severe bilateral necrosis and edema of the gray matter in the ventral horns. Moreover, the spinal cord of another dog (no. 13) exhibited severe bilateral necrosis and edema of the gray matter in association with moderate hemorrhage as well as numerous gitter cells. Six out of 9 dogs with acute SCI (No. 11, 13, 14, 15, 16, 18) showed mild multifocal axonal swellings and dilation of myelin sheaths. One dog with acute SCI (No. 19) did not show histological alterations except for few dilated myelin sheaths. In general, there were only few gitter cells in acute SCI but their number increased in the subacute phase of the disease. All dogs in the subacute group showed mild to moderate axonal swellings, dilated myelin sheaths and numerous gitter cells. The spinal cord of two dogs with subacute SCI (no. 23, 24) showed marked tissue necrosis of the white and partially of the adjacent gray matter with abundant intralesional gitter cells. Neuronal chromatolysis occurred in three dogs with acute (no. 13, 14, 17) and three dogs with subacute SCI (no. 21, 23, 24).

**Figure 1:**
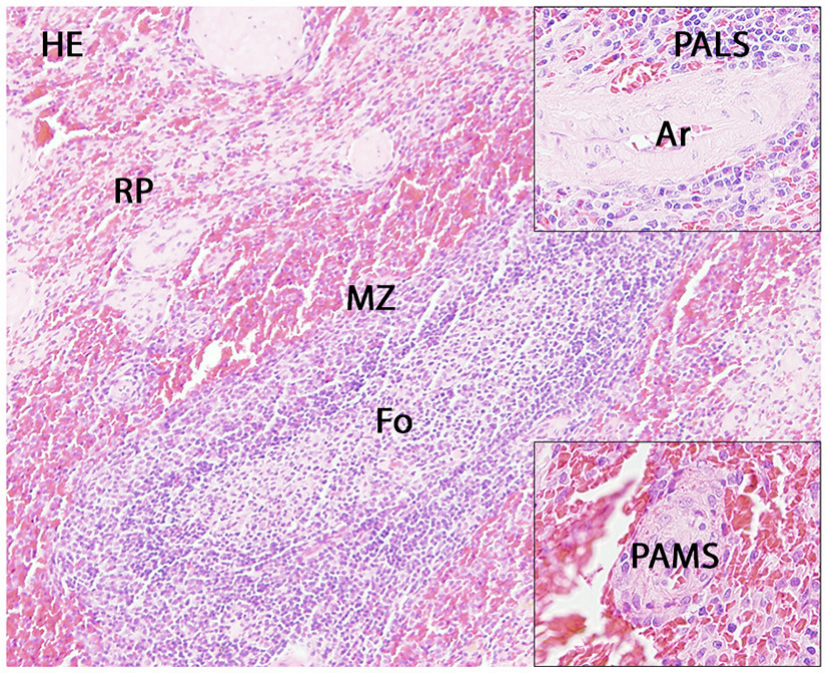
Histology of the canine spleen. Displayed are a follicle (Fo) with adjacent marginal zone (MZ) and red pulp (RP), the periarteriolar lymphoid sheath (PALS; upper insert) and the periarteriolar macrophage sheath (PAMS; lower insert). Ar: artery.

The number of spleen follicles per section ranged from 0 to 9. Extramedullary hematopoiesis was mild in dog 28, 29, and 30 and marked in dog 31 and 32. No significant lesions were present in the investigated spleens (Figure 1).

### Immunohistochemistry

In the spinal cord of control dogs (group 1), CD204-, lysozyme- and myeloperoxidase (MPO)-positive microglia/macrophages were nearly absent. Expression of BS-1, CD18, early growth response 2 (EGR2) (KROX20) and human leukocyte antigen DR (HLA, MHC-II) was generally low and very few cells, predominantly in the spinal cord gray matter, showed MAC387-staining. Ionized calcium-binding adapter molecule 1 (IBA1) was constitutively expressed throughout spinal cord gray and white matter in high numbers. CD18, EGR2, IBA1 and MHC-II were predominantly expressed by ramified microglia contrasting BS-1 and MAC387 that were expressed by round to oval cells. Immunopositive microglia/macrophages were evenly distributed throughout the gray and white matter except for MAC387.

In dogs with acute SCI (group 2), there was a significant upregulation of MAC387-positive cells in the spinal cord white matter compared to controls (group 1; *P* = 0.0236). MAC387-positive cells were scattered throughout the spinal cord white matter. In the gray matter, MAC387-positive cells were predominantly associated with areas of acute hemorrhage and tissue damage. The number of microglia/macrophages positive for BS-1, CD18, CD204, EGR2, IBA1, lysozyme, MHC-II and MPO in the spinal cord white and gray matter did not differ between control dogs and dogs with acute SCI. In dog no. 18 (gray matter necrosis), the lesion contained only few microglia/macrophages positive for BS-1, CD18, CD204, IBA1, MAC387 and MHC-II in contrast to higher numbers in the surrounding healthy tissue. Interestingly, few cells positive for lysozyme and MPO (round microglia/macrophages) were almost exclusively found within the necrotic areas but not in the adjacent tissue. There was no intralesional expression of EGR2. In dog no. 13 (gray matter necrosis with gitter cells), CD18, IBA1 and MHC-II was found in ramified, round, rod-shaped and gitter cells. BS-1 and CD204 labeled round and gitter cells. EGR2 was mainly expressed by ramified microglia but also found in few rod-shaped and round to oval microglia/macrophages as well as single gitter cells. MAC387-expressing round cells were predominantly associated with gray and white matter lesions. Similarly, lysozyme- and MPO-positive round and gitter cells were mainly found intralesionally.

**Figure 2:**
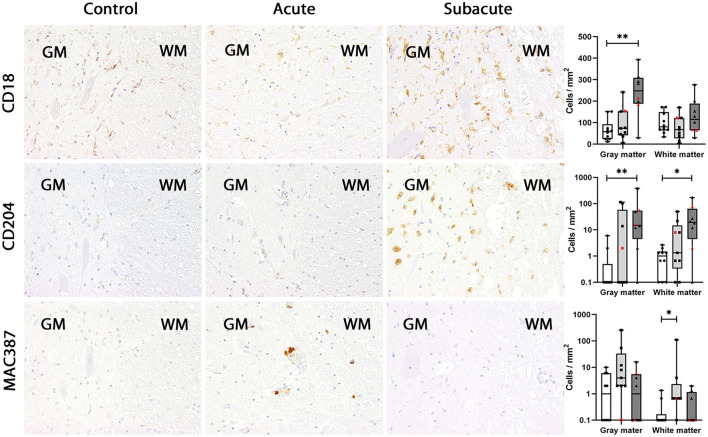
CD18, CD204 and MAC387 expression in the canine spinal cord. In control dogs, a low number of microglia/macrophages expresses CD18 in gray and white matter. CD18 expression was not changed in dogs with acute spinal cord injury (SCI) but upregulated in the gray matter in subacute SCI. Expression of CD204 was almost absent in control dogs and in dogs with acute SCI. In dogs with subacute SCI, increased numbers of CD204-positive microglia/macrophages were found in gray and white matter. In control dogs, MAC387-positive cells were rarely detected. In dogs with acute SCI, a slight increase in the number of MAC387-positive cells was found, which was significant in the white matter. In contrast, MAC387-positive cells were nearly absent in dogs with subacute SCI. Immunohistochemistry using the avidin-biotin complex immunoperoxidase method. Shown are box plots with all data points (red: cervical SCI; black: thoracolumbar SCI). Kruskal-Wallis test: *p < 0.05; **p < 0.01.

**Figure 3:**
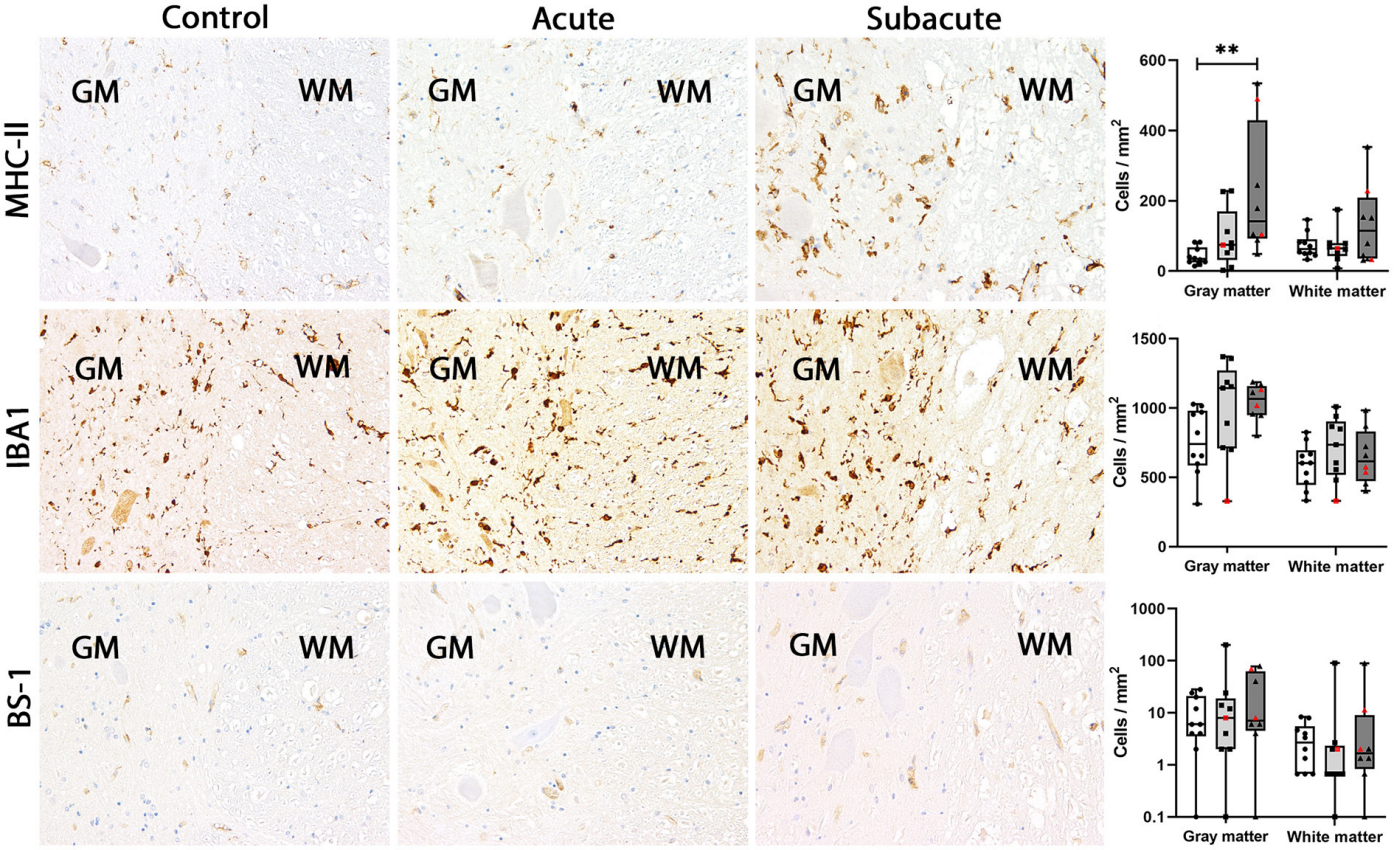
MHC-II, IBA1 and BS-1 expression in the canine spinal cord. In control dogs, low numbers of microglia/macrophages expressed MHC-II in gray and white matter. No change in MHC-II expression was found in dogs with acute spinal cord injury (SCI). In dogs with subacute SCI, the number of MHC-II-positive cells increased significantly in gray matter only. IBA1 is expressed by a high number of microglia/macrophages in gray and white matter of control dogs. This number did not change significantly in dogs with acute and subacute SCI. Only few BS-1-positive cells were present in gray and white matter of control dogs. This number stayed at low levels in dogs with acute and subacute SCI. Immunohistochemistry using the avidin-biotin complex immunoperoxidase method. Shown are box plots with all data points (red: cervical SCI; black: thoracolumbar SCI). Kruskal-Wallis test: **p < 0.01.

**Figure 4:**
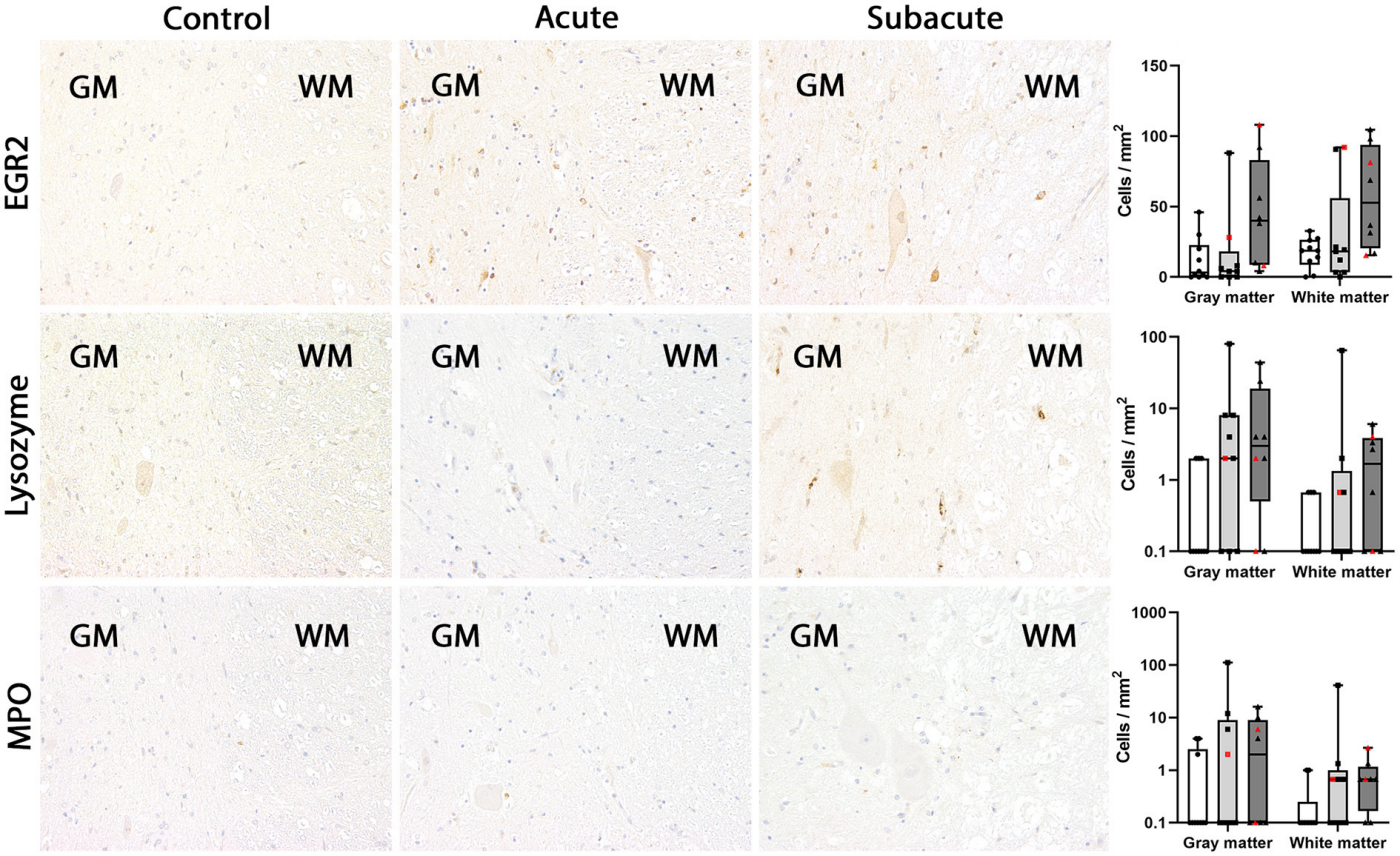
EGR2, lysozyme and myeloperoxidase (MPO) expression in the canine spinal cord. In control dogs, EGR2-, lysozyme- and MPO-positive microglia/macrophages were rarely detected in gray and white matter. The numbers of immunopositive cells did not change significantly in dogs with acute and subacute spinal cord injury (SCI). In dogs with subacute SCI, higher numbers of EGR2-positive cells were found in gray and white matter of several dogs but significant difference between the groups was lacking. MPO-positive cells are not illustrated in this figure due to the extremely low number. Immunohistochemistry using the avidin-biotin complex immunoperoxidase method. Shown are box plots with all data points (red: cervical SCI; black: thoracolumbar SCI).

**Figure 5:**
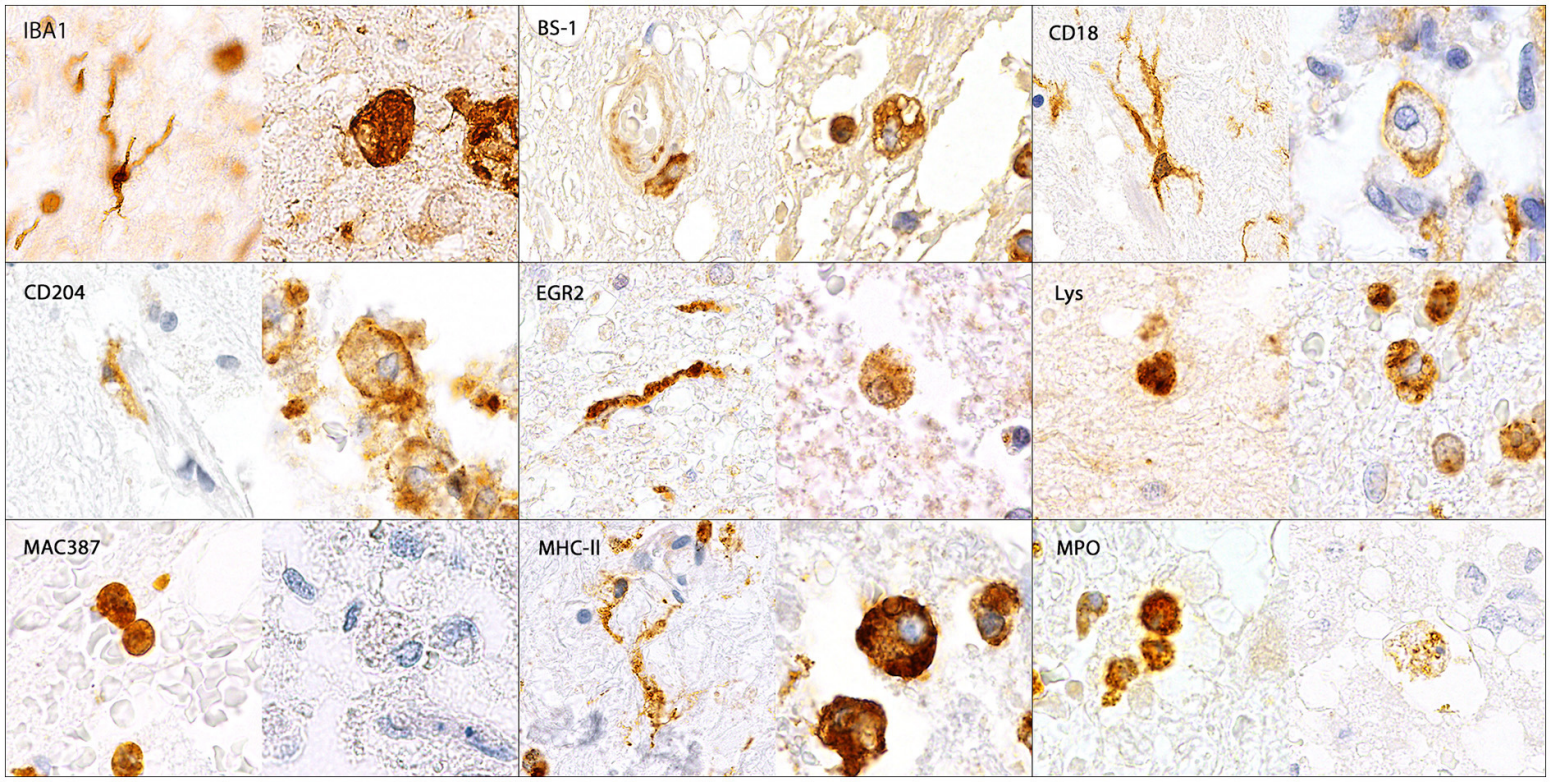
Illustration of IBA1-, BS-1-, CD18-, CD204-, EGR2-, lysozyme (Lys)-, MAC387-, MHC-II- and myeloperoxidase (MPO)-immunopositive microglia/macrophages in the canine spinal cord. IBA1, CD18, CD204, EGR2 and MHC-II were expressed by ramified (arrows), rod-shaped (arrowheads), round, amoeboid and gitter cells (asterisks). In contrast, BS-1, Lys and MPO were only detected in round, amoeboid and gitter cells but not in ramified or rod-shaped microglia. MAC387 was only expressed by medium-sized round cells. Immunohistochemistry using the avidin-biotin complex immunoperoxidase method.

In dogs with subacute SCI (group 3), CD18, CD204 and MHC-II were markedly upregulated in the spinal cord gray matter (group 3 vs. group 1: CD18: *P* = 0.0054; CD204: *P* = 0.0081; MHC-II: *P* = 0.0036). In the white matter, more CD204-positive microglia/macrophages were found in subacute SCI compared to controls (*P* = 0.0186). Though the absolute number of EGR2-positive microglia/macrophages was higher in dogs with subacute SCI (group 3) compared to control dogs (group 1) and dogs with acute SCI (group 2) in both white and gray matter, no significant difference was found [group 3 vs. group 1: *p* = 0.0588 (gray matter); *p* = 0.0837 (white matter)]. CD18-, CD204, EGR2 and MHC-II were found in ramified, rod-shaped, round and gitter cells throughout the whole section. Finally, there were no significant differences between the groups in the number of BS-1-, IBA1-, lysozyme- and MPO-positive microglia/macrophages in the spinal cord white and gray matter. BS-1 labeling occurred mainly in round microglia/macrophages or gitter cells often in close vicinity to blood vessels (Figures 2–5).

**Figure 6:**
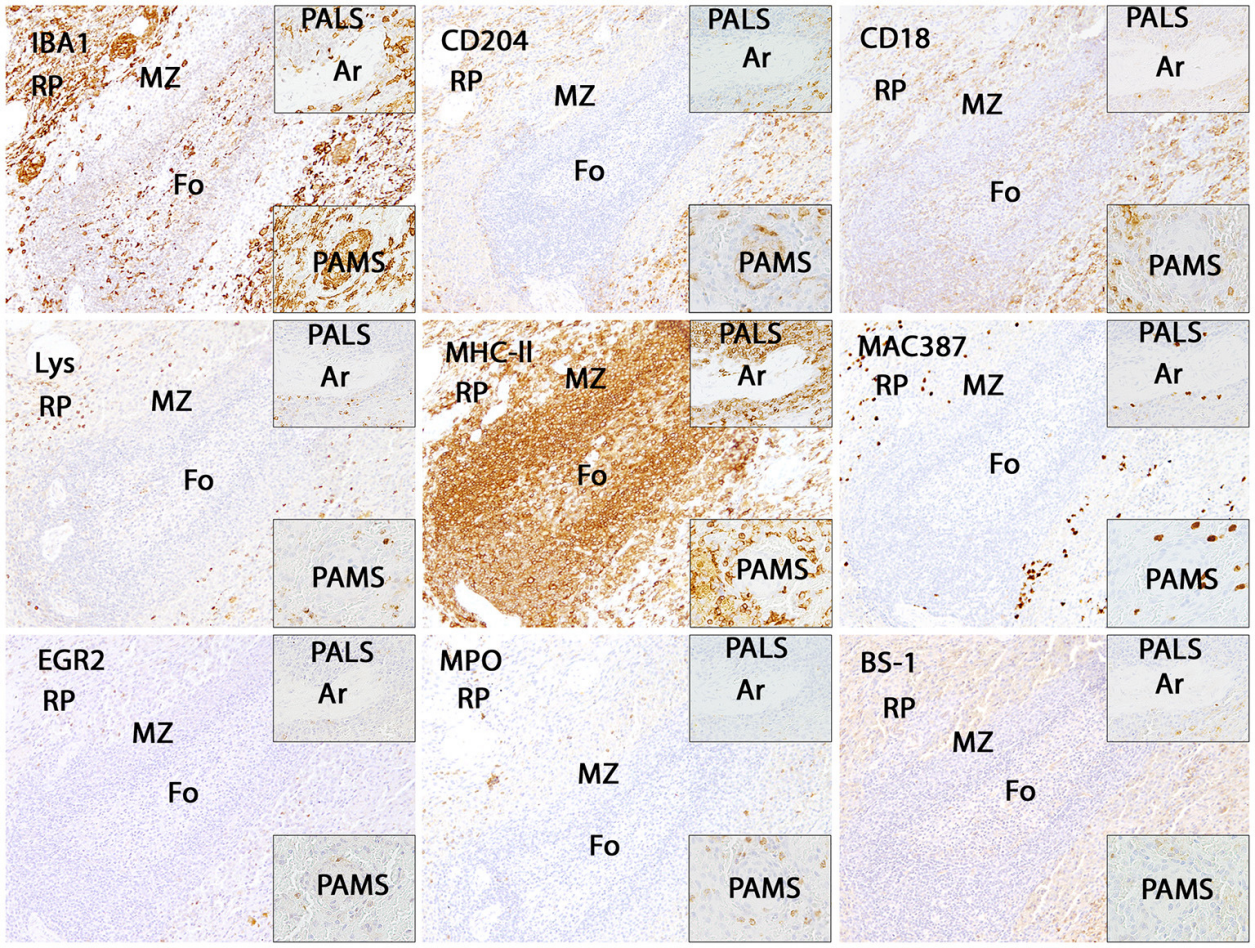
Illustration of IBA1, CD204, CD18, lysozyme (Lys), MHC-II, MAC387, EGR2, myeloperoxidase (MPO) and BS-1 immunostaining in the canine spleen. The white pulp including follicles (Fo), marginal zone (MZ) and periarteriolar lymphocytic sheath (PALS; upper insert) is characterized by intense expression of MHC-II, whereas all other markers are expressed by only few cells. Within the periarteriolar macrophage sheath (PAMS; lower insert) numerous cells are positive for IBA1 and CD204. The red pulp contains moderate to high numbers of IBA1-, CD204-, CD18- and MHC-II-positive cells. Immunohistochemistry using the avidin-biotin complex immunoperoxidase method. Ar: artery.

**Figure 7:**
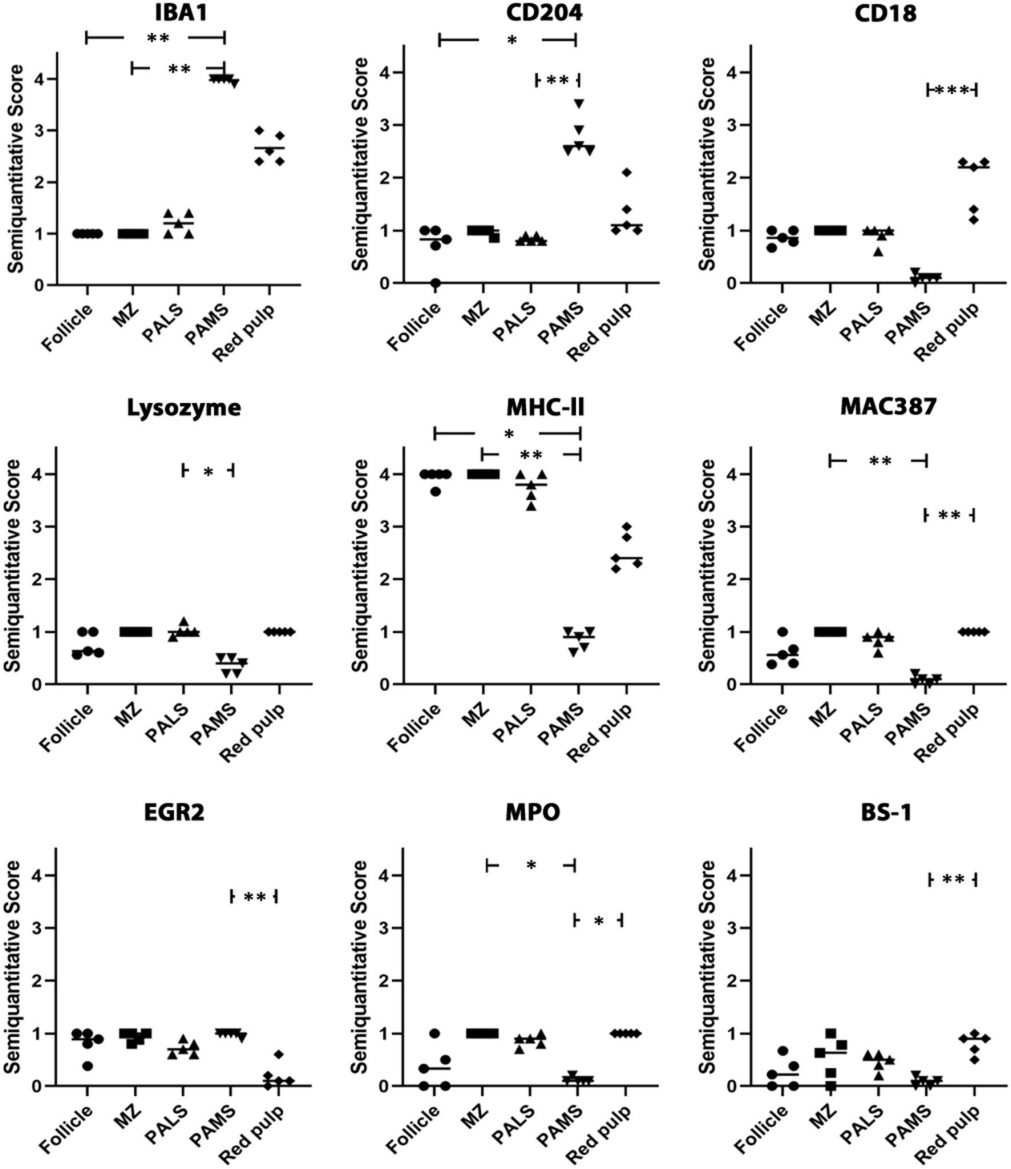
Analysis of IBA1, CD204, CD18, lysozyme, MHC-II, MAC387, EGR2, myeloperoxidase (MPO) and BS-1 expression in the canine spleen. The white matter of the spleen including follicles, marginal zone (MZ) and periarteriolar lymphocytic sheath (PALS) contains abundant cells expressing MHC-II, whereas cells expressing other markers are rare. The majority of cells in the periarteriolar macrophage sheath (PAMS) are positive for IBA1 and CD204 but negative for other markers. Several cells in the red pulp of the spleen express IBA1, CD204, CD18 and/or MHC-II, whereas only few cells express other markers. Scatter plots of semiquantitative scores (0 = 0%; 1 = 1–25%; 2 = 26–50%; 3 = 51–75%; 4 = 76–100% positive cells). The bars indicate mean values. Friedman-test: *p < 0.05; **p < 0.01; ***p < 0.001.

In the white pulp of the spleen of dogs of group 4 (follicle, marginal zone and PALS) a high MHC-II expression (MHC-II-positive cells included macrophages, dendritic cells, T cells and B cells) was found, whereas expression of other markers was generally low. The PAMS were characterized by high expression of IBA1 and CD204, low expression of EGR2, MHC-II and lysozyme and almost lacking expression of MAC387, CD18, MPO and BS-1. In the red pulp, about half of the nucleated cells stained positive for CD18, IBA1 and MHC-II. All other markers were expressed on a low level within the red pulp and only single cells stained positive for EGR2 (Figures 6, 7; Table 3).

**Table 3:** Marker expression within follicles, marginal zone, the periarteriolar lymphoid sheath (PALS), the periarteriolar macrophage sheath (PAMS) and the red pulp of the canine spleen.

**Marker**	**Follicle**	**Marginal zone**	**PALS**	**PAMS**	**Red Pulp**
**BS-1**	-/+	+	-/+	-/+	+
**CD18**	+	+	+	-/+	++
**CD204**	+	+	+	+++	+
**EGR2**	+	+	+	+	-/+
**IBA1**	+	+	+	++++	+++
**Lysozyme**	+	+	+	-/+	+
**MAC387**	-/+	+	+	-/+	+
**MHC-II**	++++	++++	++++	+	+++
**MPO**	-/+	+	+	-/+	+

Follicles, marginal zone and PALS are characterized by strong MHC-II expression due to the high number of immunopositive B and T cells in this area, whereas other markers are rarely expressed. Most cells in the PAMS express IBA1 and CD204 but not BS-1, CD18, lysozyme, MAC387 and/or MPO. Within the red pulp, the majority of cells stains positive for CD18, IBA1 and MHC-II, whereas most cells are negative for BS-1, CD204, EGR2, lysozyme, MAC387 and MPO.

-: no immunopositive cells; +: < 25% immunopositive cells; ++: 26–50% immunopositive cells; +++: 51–75% immunopositive cells; ++++: >75% immunopositive cells.

## Discussion

The present study investigated the immunophenotype of microglia/macrophages in the spinal cord of dogs with SCI. Microglia/macrophages in the control spinal cord are characterized by expression of IBA1, whereas only few cells exhibit BS-1, CD18, EGR2 and MHC-II. CD204, lysozyme, MAC387 and MPO are almost absent in these cells. In dogs with acute SCI, the number of MAC387-positive cells increased significantly within the white matter. MAC387 staining was confined to medium-sized round cells, which most likely represented recently infiltrating macrophages. Only few lysozyme and MPO-positive cells, mainly medium-sized round cells and few gitter cells, were found in the injured spinal cord predominantly associated with hemorrhage and necrosis. No major difference in microglia/macrophage immunophenotype was found between control dogs and dogs with acute SCI corresponding to few histological lesions. Gitter cells were mainly present in dogs with subacute SCI and characterized by expression of all markers except for MAC387. In dogs with subacute SCI, marker expression of microglia/macrophage also differed between the gray and the white matter of the spinal cord. Increased numbers of microglia/macrophages expressed CD18, CD204 and MHC-II during subacute SCI within the gray matter, but only CD204 was significantly upregulated within the white matter. Macrophage-rich domains (PAMS) of the canine spleen only show a high expression of CD204 and IBA1.

Microglia/macrophages in the spinal cord of control dogs, mostly representing ramified microglia, are characterized by constitutive expression of IBA1 and low expression of other markers. IBA1 is a highly conserved cytoplasmic protein that functions in cell migration, phagocytosis and signaling (63–65). It is expressed by almost all subsets of the monocytic/macrophage lineage (66). MHC-II is upregulated in microglia/macrophages upon activation (38, 67) but weak expression was also observed in the non-diseased CNS of dogs, rodents and humans (19, 23, 67, 68). MPO expressing cells were almost absent in the control spinal cord. Correspondingly, studies using flow cytometry showed that microglia isolated of healthy dog brains only generates a low MPO activity even after stimulation (29, 69). Nevertheless, another flow cytometry study showed MPO activity in a high percentage of canine microglia derived from the healthy canine brain and spinal cord (60), which might be related to a higher sensitivity of flow cytometry in enzyme detection compared to immunohistochemistry. Moreover, the process of isolating microglia for analysis might have induced a low MPO activity in the cells.

Within the white matter of dogs with acute SCI an increased number of MAC387-positive cells was observed similar to a previous study (19). MAC387 is a potent marker for blood-borne macrophages and not expressed by human microglia *in vitro* (70–73). Correspondingly, in canine spinal cord slice cultures no MAC387-expression was detected by microglia cells up to 9 days post insult (19). Thus, spinal cord white matter lesions are initially infiltrated by blood-borne macrophages most likely reacting to the acute traumatic insult. Interestingly, studies in human cell cultures showed that its expression declines with macrophage maturation after 2–3 days (74). Thus, low numbers of MAC387-positive cells in dogs with subacute SCI might be caused by differentiation of CNS-entering monocytes and loss of MAC387-expression making them indistinguishable from the resident population (75–77), but information about “turn-over” kinetics in dogs is lacking.

The expression of BS-1, EGR2, IBA1, lysozyme and MPO was similar in control and injured spinal cord. Low levels of lysozyme-positive microglia/macrophages indicate that this molecule plays an ancillary role in the pathogenesis of SCI. Similar to the low number of MPO-positive cells in dogs with acute and subacute SCI, a previous flow cytometry study showed no upregulation in the percentage of microglia displaying MPO activity after SCI (44). A study conducted in MPO-knock out and wild-type mice measuring the level of its reaction product hypochlorous acid (HOCl) indicated that MPO contributes to secondary injury during SCI, but HOCl concentration peaked around 12 hours post injury and decreased afterwards. Moreover, high levels of HOCl coincided with neutrophilic influx suggesting that HOCl is mainly generated by neutrophils during SCI, whereas prominent microglia proliferation did not result in extensive HOCl production (78). Consequently, low numbers of MPO expressing cells might be related to the time point of our investigation as the earliest changes measured were at 24 h post injury and/or to lower numbers of neutrophils in dogs compared to mice.

IBA1 is constitutively expressed by microglia/macrophages (66, 79–81). Following activation, IBA1 expression in microglia is enhanced (82), though a rat study using experimental SCI showed IBA1 expression only in a subset of phagocytic active cells peaking around day 3 post injury (83). In the present study, the total number of IBA1-positive cells only increased slightly toward the acute phase of SCI without reaching significance. These inconsistent observations might be caused by species-specific variations in IBA1 expression and/or be related to the antibodies used in the respective studies, as in the rat study a monoclonal mouse antibody and in the present study a polyclonal rabbit antibody was utilized. Moreover, application of dexamethasone induced downregulation of IBA1 during inflammatory conditions in previous studies (84). As 8 out of 17 dogs with SCI received corticosteroids and/or NSAID, treatment might have prevented increased IBA1 expression in spinal cord lesions of the dogs in the current study.

Increased numbers of CD18-, CD204- and MHC-II-positive microglia/macrophages were found in dogs with subacute SCI. Canine microglia are known to express the β2-integrin CD18 (29, 69, 85), which is part of the complement receptors CR3 (CD11b/CD18, Mac-1) and CR4 (CD11c/CD18) (86). The heterodimer CD11a/CD18 (lymphocyte function-associated antigen, LFA-1) is an adhesion molecule involved in leucocyte extravasation (86). Consequently, upregulation of CD18 in canine microglia/macrophages plays a central role in inflammatory processes and removal of cellular debris during the repair of spinal cord lesions (39). Moreover, CR3 upregulation has been reported in the regenerating facial nucleus in rats and due to the lack of phagocytosis an additional role for this molecule has been proposed in tissue repair (87). CD204 (macrophage scavenger receptor 1, Msr1) is also able to recognize a wide range of endogenous and exogenous ligands, functions in cell adhesion and is involved in the clearance of apoptotic cells (88). Interestingly, a recent study found elevated TNFα and IL-6 levels in the serum and spleen of mice lacking CD204-positive macrophages predisposing to septic shock (89). Likewise, CD204 has been described as marker for anti-inflammatory macrophages and thus likely contributes to the resolution of inflammatory processes. However, CD204 seems to have both, beneficial and deleterious roles in various pathologic conditions (88), thus the role of CD204 in SCI needs to be clarified in further studies. Previous studies also described an upregulation of microglial expression of MHC-II during SCI in dogs (44) especially in the subacute phase of SCI (19). Similarly, an upregulation of MHC-II was detected in the murine spinal cord after trauma (22) as well as in human SCI (23). One study also showed a higher expression of MHC-II in the cervical compared to the thoracolumbar segments of dogs after SCI (44), whereas marker expression of cervical and thoracolumbar segments was similar in the present study. Increased numbers of microglia/macrophages expressing CD18, CD204 and MHC-II in dogs with subacute SCI suggest for enhanced antigen recognition, processing and presentation, as well as cell migration and phagocytosis during this stage. High numbers of gitter cells in subacute SCI underline the resorptive nature of the inflammation at the injury site. Thus, the results of the present study support the hypothesis that microglia/macrophages contribute to a regeneration-promoting milieu by removal of regeneration-inhibiting myelin components in naturally occurring SCI in dogs. However, the role of microglia/macrophages during SCI is regarded a double-edged sword (31, 37, 44). While the clearance of myelin debris from the injury site could promote axonal regrowth and functional recovery (39, 40), the excessive secretion of cytokines, MMPs and ROS may contribute to bystander damage aggravating initial traumatic lesions (27, 49–51).

It is remarkable that upregulation of CD18 and MHC-II only reached significance in the gray but not in the white matter partially mirroring previous findings in humans and rodents (22, 23). This upregulation in the gray matter might be related to phagocytosis of presynaptic terminals of interrupted descending tracts distal to the site of axonal injury in early stages of SCI, whereas the removal of degenerated fibers occurs later (90, 91). Furthermore, variations in the microglia expression pattern between white and gray matter might be related to intrinsic differences in their transcriptional profiles as previously described in the human brain (92). Human microglia showed a higher expression of type I interferon genes in the gray matter, whereas microglia located in the white matter exhibited a higher expression of NF-κB pathway genes. Nevertheless, despite the importance of microglia in the immune response of the CNS (37, 60, 69, 73, 93), the significance of its antigen-presenting capacity during canine and human SCI is questionable due to the lack of an adequate T-cell response (19, 23). It is known that phagocytosis of myelin and cellular debris induces MHC-II-expression (94) but an upregulation of MHC-II after various types of injury without subsequent antigen-presentation has been reported (31, 95, 96). An impaired T-cell-response may prevent the spinal cord from further secondary damage, thus indicating a protective function (23).

In contrast to microglia during SCI, functions of different histiocytic cell types in the spleen such as dendritic cells and macrophages are well known. Therefore, we also investigated the expression of our markers within the canine spleen to facilitate the interpretation of the microglia/macrophage phenotype found in the lesioned spinal cord. The white pulp (including follicle and PALS) was mainly characterized by high MHC-II expression, which is related to antigen presentation. Moreover, not only macrophages, dendritic cells and B-cells, but also T-cells of various species including humans, cattle, horses, rats and dogs (not mice) express MHC-II molecules, which explains high MHC-II expression in the splenic white pulp (97). PAMS are predominantly composed of macrophages (98) and correspondingly showed a strong IBA1 and CD204 expression. While IBA1 fails to discriminate between macrophages and dendritic cells (99, 100), expression of CD204 in dendritic cells of different species seems to be variable (101, 102) and canine dendritic cells do not express CD204 under non-diseased conditions (103). Thus, prominent expression of CD204 in PAMS indicates low numbers of dendritic cells at this site. CD204 most likely acts as a pattern recognition receptor (104, 105) and thereby contributes to the clearing of the blood from potentially harmful substances and aged erythrocytes in PAMS, which surround arterial capillaries in the spleen (98). Expression of other markers was low (EGR2, lysozyme, MHC-II) or almost absent (BS-1, CD18, MAC387, MPO) within the PAMS indicating that these markers are not regularly expressed by histiocytic cells. Renewal of the macrophage sheath is also largely maintained by local proliferation corresponding to the lack of MAC387-positive cells (106). In the red pulp, a high number of cells were positive for CD18, IBA1 and MHC-II. CD18 is not exclusively expressed by tissue macrophages, but also by all bone-marrow derived leukocytes (86). Due to the presence of extramedullary hematopoiesis, immunopositive cells found in the red pulp surely included lymphocytes, plasma cells and different hematopoietic cells in contrast to the spinal cord. Nonetheless, these findings indicate that under physiologic conditions different macrophage phenotypes can be found in the compartments of the canine spleen. Additional markers might help to picture microglia/macrophage phenotype in more detail. The selected markers are appropriate for microglia/macrophage staining in the CNS but more cells react with them within the canine spleen. Therefore, the comparison of microglia/macrophage phenotype inside and outside the CNS should be performed carefully. Moreover, functional aspects of the investigated markers during SCI remain to be specified in further studies.

In conclusion, the results of the present study shows a time-dependent acquisition of different microglia/macrophage phenotypes in the white and gray matter of the injured spinal cord in dogs. During subacute canine SCI, microglia/macrophage express molecules involved in cell adhesion, migration and phagocytosis and seem to acquire a scavenging phenotype, similar to splenic PAMS-associated macrophages under healthy conditions. In contrast to PAMS-associated splenic macrophages, CNS microglia/macrophage express high levels of the β2-integrin CD18 during SCI possibly related to the migration of these cells to white and gray lesions. The production of MPO seems to play a subordinate role in the pathogenesis of spinal cord lesions in dogs but further studies are needed to elucidate the interactions of microglia/macrophages with other resident and infiltrating immune cells during the acute and subacute phase of canine SCI in detail. Observations in canine SCI might also facilitate the transition of findings obtained from experimental rodent models into clinical trials.

## Data availability statement

The original contributions presented in the study are included in the article/supplementary material, further inquiries can be directed to the corresponding author.

## Ethics statement

The animal study was reviewed and approved by Niedersächsisches Landesamt für Verbraucherschutz und Lebenmittelsicherheit, Oldenburg, Germany. Written informed consent was obtained from the owners for the participation of their animals in this study.

## Author contributions

BK performed and analyzed the immunohistochemical stainings, generated the figures and tables, and drafted the manuscript. MB performed the statistical analysis. WB generated the idea of the study, performed data analysis, and edited the manuscript. IG analyzed data, designed figures and tables, and edited the manuscript. All authors contributed to the article and approved the submitted version.

## Funding

This Open Access Publication was funded by the Deutsche Forschungsgemeinschaft (DFG, German Research Foundation) - 491094227 “Open Access Publication Funding” and the University of Veterinary Medicine Hannover, Foundation.

## Conflict of interest

The authors declare that the research was conducted in the absence of any commercial or financial relationships that could be construed as a potential conflict of interest.

## Publisher's note

All claims expressed in this article are solely those of the authors and do not necessarily represent those of their affiliated organizations, or those of the publisher, the editors and the reviewers. Any product that may be evaluated in this article, or claim that may be made by its manufacturer, is not guaranteed or endorsed by the publisher.
